# Classification of T lymphocyte motility behaviors using a machine learning approach

**DOI:** 10.1371/journal.pcbi.1011449

**Published:** 2023-09-11

**Authors:** Yves Carpentier Solorio, Florent Lemaître, Bassam Jabbour, Olivier Tastet, Nathalie Arbour, Elie Bou Assi

**Affiliations:** 1 Centre de Recherche du CHUM (CRCHUM), Montréal, Québec, Canada; 2 Department of Neuroscience, Université de Montréal, Montréal, Québec, Canada; University College London, UNITED KINGDOM

## Abstract

T lymphocytes migrate into organs and interact with local cells to perform their functions. How human T lymphocytes communicate with organ-specific cells and participate in pathobiological processes remains unresolved. Brain infiltration of T lymphocytes is associated with multiple neurological disorders. Thus, to characterize the behavior of human T lymphocytes reaching the human brain, we performed time-lapse microscopy on human CD8^+^ T lymphocytes co-cultured with either primary human astrocytes or neurons. Using traditional manual and visual assessment of microscopy data, we identified distinct CD8^+^ T lymphocyte motility behaviors. However, such characterization is time and labor-intensive. In this work, we trained and validated a machine-learning model for the automated classification of behaviors of CD8^+^ T lymphocytes interacting with astrocytes and neurons. A balanced random forest was trained for the binary classification of established classes of cell behaviors (synapse vs. kinapse) as well as visually identified behaviors (scanning, dancing, and poking). Feature selection was performed during 3-fold cross-validation using the minimum redundancy maximum relevance algorithm. Results show promising performances when tested on a held-out dataset of CD8^+^ T lymphocytes interacting with astrocytes with a new experimenter and a held-out independent dataset of CD8^+^ T lymphocytes interacting with neurons. When tested on the independent CD8^+^ T cell-neuron dataset, the final model achieved a binary classification accuracy of 0.82 and a 3-class accuracy of 0.79. This novel automated classification approach could significantly reduce the time required to label cell motility behaviors while facilitating the identification of interactions of T lymphocytes with multiple cell types.

## Author summary

T lymphocytes are immune cells that enter into organs and then communicate with local cells to perform their functions. The mechanisms of such interactions, especially in the case of pathobiological processes, remain unclear. Indeed, multiple neurological disorders are characterized by an infiltration of T lymphocytes into the brain and spinal cord. Time-lapse microscopy allows to observe microscopic cellular dynamics over extended periods of time. We used this technique to characterize the behavior of T lymphocytes communicating with human brain cells (neurons and astrocytes). While manual and visual assessment of the recorded videos allowed to identify distinct behaviors of T lymphocytes, it is time and labor consuming. To automate the classification of T lymphocytes’ behaviors, we developed a machine learning model based on features extracted from time-lapse microscopy videos. We optimized the model by selecting the best features. The classification model was based on a balanced random forest, a combination of multiple decision trees. We trained and tested the model on 2 distinct datasets of T lymphocytes interacting with astrocytes and neurons. We found promising performances highlighting that the proposed model can automate the labeling of T lymphocyte behaviors dialoguing with human brain cells.

## 1. Introduction

Immune cells patrol our bodies for cues to deploy their functions [1]. Even though multiple signals, such as chemokines and cell adhesion molecules, have been characterized, the mechanisms driving immune cell migration and motility still need to be resolved. Moreover, numerous pathological conditions are associated with an enhanced tissue infiltration of lymphocytes. For example, an elevated number of T lymphocytes has been observed in the central nervous system (CNS) of almost all neurodegenerative diseases [2]. Therefore, a better understanding of the immune patrol is essential to unravel critical immunological responses, especially in the CNS.

*In vivo* intra-vital and *in vitro* time-lapse microscopy allows the investigation of immune cells’ dynamic interactions, such as T lymphocytes, with other cell types at a single-cell resolution. When activated T lymphocytes encounter other cells harboring the cognate complex recognized by their T cell receptor [3], they decelerate. Upon a strong recognition, T lymphocytes arrest and establish a long-lasting interaction with the encountered cells, referred to as a synapse [4–6]. The immune synapse triggers multiple responses in T lymphocytes, including cytotoxic functions and cytokine production. Alternatively, following a weak recognition, T lymphocytes exhibit higher motility and transient contact with the encountered cells resulting in a more dynamic interaction referred to as a kinapse [5,6].

A growing body of evidence demonstrates that both the immune system and the CNS exhibit species differences [7–9]. Therefore, to specifically investigate human T lymphocyte-neural cell interactions, we developed a co-culture system capturing the dynamic interactions between human CD8^+^ T lymphocytes and cells from the human CNS, primary human astrocytes and neurons [10]. Using time-lapse microscopy, we imaged CD8^+^ T lymphocytes interacting with these abundant human neural cells [10]. Based on spatiotemporal variables and cellular morphology, we described different types of interactions presenting specific characteristics associated with either synapse or kinapse-like behavior. Notably, exposition to inflammatory cytokines or pathological conditions had an impact on the proportions and the spatiotemporal characteristics of these different behaviors [10–12]. However, cell motion classification was performed manually and was time-consuming. Moreover, we cannot rule out experimenter-induced bias, thus reducing the robustness of our assays.

**Fig 1:**
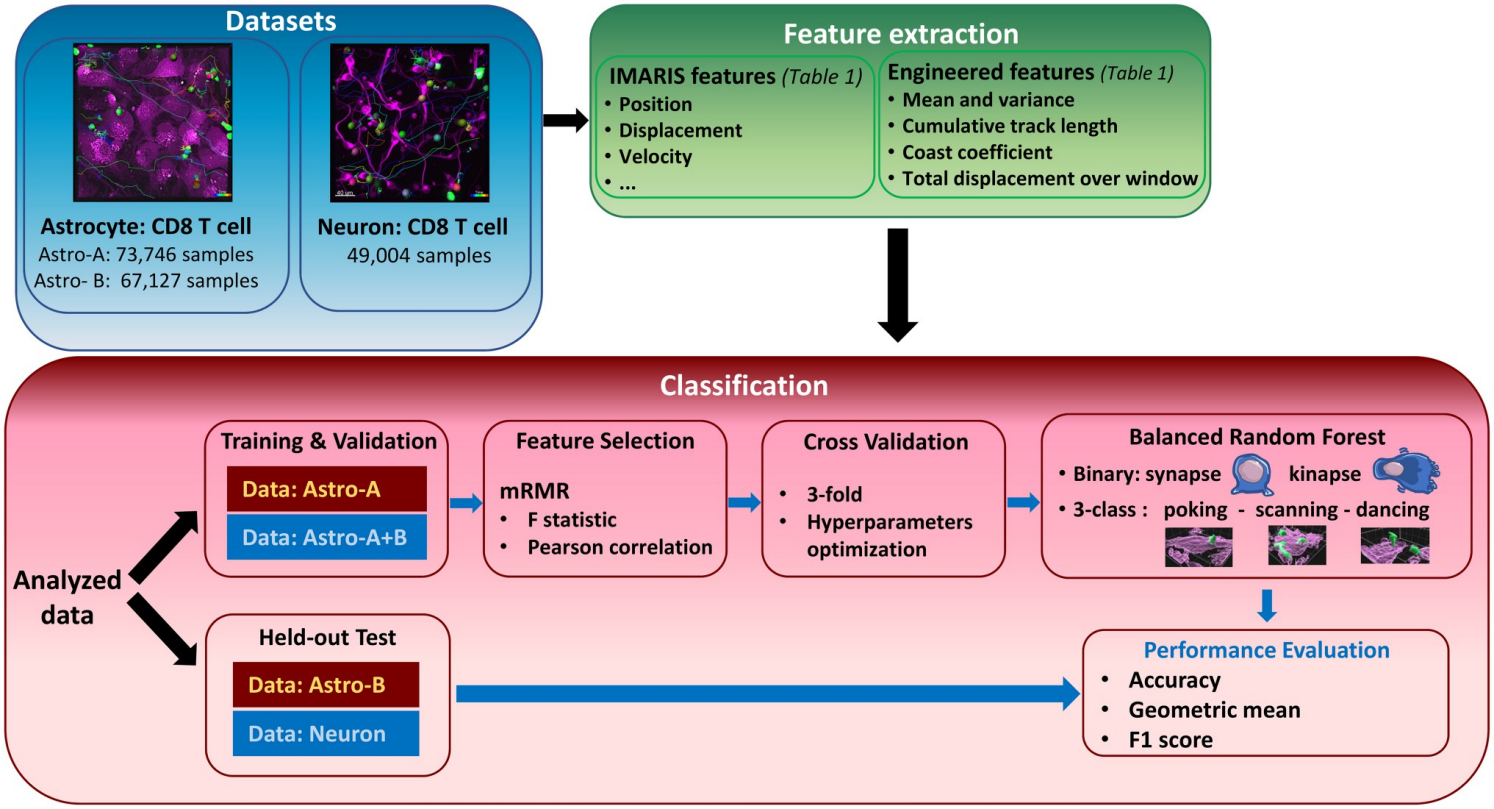
Framework of data analysis and model construction. The first classifier was trained and optimized on the Astro-A dataset (red box) and tested on the Astro-B dataset whereas the second classification was performed by using the combined Astro A+B datasets (blue box) for training and validation and the Neuron dataset for testing. The balanced random forest model was trained for the binary (synapse vs. kinapse-like behaviors) and the 3-class (poking, scanning, dancing) classification.

Our main objective was to develop a machine-learning model capable of automatically classifying CD8^+^ T lymphocyte behaviors while interacting with primary human astrocytes and neurons. A balanced random forest classifier was trained using features extracted from the *Imaris* software, broadly used by the scientific community to perform time-lapse microscopy image analysis. We developed a model and validated it on the first dataset of CD8^+^ T lymphocytes co-cultured on human astrocytes (Astro dataset). The classifier was first trained and cross-validated (3-fold) on data from one experimenter (Astro A) and tested on held-out data from a second experimenter (Astro B). The classifier was then trained and cross-validated on data from both experimenters (Astro dataset) and tested on a held-out dataset of CD8^+^ T lymphocytes interacting with human neurons (Neuron dataset) (Fig 1). Based on our novel approach, we propose a classification model of CD8^+^ T lymphocyte motility patterns that could be extended to other immune cells in contact with diverse cell types. This machine learning-based tool will ease and accelerate the identification of normal or dysfunctional interactions of T lymphocytes with targeted cells in physiological and pathological contexts or using drug or immune therapy strategy. It provides a computational approach to characterize and compare cell behaviors across several conditions of interest.

## 2. Materials and methods

### 2.1 Ethics statement

All required ethical guidelines were followed. These studies were approved by the Centre Hospitalier de l’Université de Montréal (CHUM) ethics boards (BH07.001, HD07.002). Fetal brain tissue was obtained after written informed consent (ethical committee of CHU Sainte-Justine, Montreal QC, Canada, CER#2126; University of Washington Birth Defects Research Laboratory Seattle, Washington, USA, STUDY00000380). All donors gave written informed consent for blood donation in accordance with the local ethical committee.

### 2.2 Isolation, culture, and labeling of human astrocytes and neurons

Primary human astrocyte cultures were isolated from human fetal brain tissues as previously detailed [10]. Astrocytes were labeled with Orange-CMRA dye, plated, and cultured as previously described [10]. Primary human neurons were isolated from human fetal brain as previously published [10]. Neurons were labeled with Taxol Janelia Fluor 646 before imaging [10]. Prior to the addition of CD8^+^ T lymphocytes, experimenter A exposed astrocytes or neurons to tumor necrosing factor (TNF) and interferon-gamma for 48 hours [11], while experimenter B exposed these cells to interleukin-27 or interleukin-1β for 24h [10,12].

### 2.3 CD8^+^ T cell isolation, activation, and labeling

Peripheral blood mononuclear cells (PBMC) were isolated using Ficoll density gradient as previously described [10]. CD8^+^ T lymphocytes were positively isolated using CD8 Microbeads (Milteniy Biotech) according to the manufacturer’s instructions. Experimenter A activated CD8^+^ T lymphocytes overnight on anti-CD3 pre-coated plates in the presence of soluble anti-CD28 antibodies and recombinant human interleukin-15 as previously published [11]. Experimenter B activated CD8^+^ T lymphocytes on anti-CD3 pre-coated wells in the presence of anti-CD28 antibodies. Activated CD8^+^ T cells were harvested and labeled with carboxyfluorescein succinimidyl ester as previously published [10,11].

### 2.4 In vitro live imaging co-culture

CFSE-labeled CD8^+^ T lymphocytes were added to astrocytes (T cell: astrocyte ratio, 4:1) or neurons (T cell:neuron ratio 10:7) as previously described [10,11]. Co-cultures were imaged for 2h (1 frame/min) using the spinning-disc microscopy settings previously described [10]. Image analysis, post-acquisition processing and CD8^+^ T lymphocyte tracking using the *Imaris* software (V9.6 Bitplane, Oxford Instruments group) were performed as previously described [10]. Tracks exhibiting less than 300s (5 time points) duration in the imaged field were excluded. All cell tracks were manually corrected using the manual editor when misdetection or non-detection were observed.

### 2.5 Dataset format

Three distinct datasets were used in this project. The first two datasets were obtained from the analysis of astrocytes-CD8^+^ T cell co-cultures from two different experimenters (Astro A and Astro B datasets) (Fig 1). The third dataset was acquired by merging analyzed neuron-CD8^+^ T cell co-culture experiments from the two experimenters (Neuron dataset). Numerical features of each cell track were exported from *Imaris* as .csv files containing cell statistics along with extra information (track id, donor id, disease, treatment, experiment), and CD8^+^ T cell behavior. Each line of the .csv file corresponded to a given cell at a specific time step, with an interval of 1 min between successive measures. A custom R script was then used to merge the tracks for each dataset into one .csv file and drop all channel-related parameters. We previously classified CD8^+^ T lymphocytes into four behaviors in our original publications [10,11]: scanning, dancing, poking and round. The distinction between round and poking cells was based on morphological appearance. Since our AI approach cannot perform such distinction, we combined poking and round behaviors as poking behavior. Our analysis included three visually identified behaviors: scanning, dancing, and poking. We performed the binary classification based of established classes of behaviors (synapse vs. kinapse).

### 2.6 Feature extraction

A total of 90 features were extracted from the time-lapse microscopy files allowing us to characterize CD8^+^ T cell motion over time. The first set of features (29 features) was extracted using the *Imaris* software (Table 1). Additional 61 features were proposed in this work (Table 1). We previously observed that CD8^+^ T lymphocytes could change from one behavior to another during the 2-hour acquisition [10]. To capture these behavior changes in our analysis, we segmented the original cell tracks into shorter-duration tracks. Such an approach allowed us to refine our analysis at the single behavior level rather than at the single cell level. We excluded tracks that were less than 5 min from the original analysis; some cells rapidly escaped the filmed frame. We selected a moving window approach with a 5-minute track duration. The window was centered on the corresponding time point, and successive windows moved by one step (1 min) at a time. For the time points at the beginning and end of a sequence for a given cell, the first and last computed values were copied over, respectively.

**Table 1:** List of features extracted and engineered from *Imaris*.

Features	Characteristics	Number of features
Acceleration	X, Y, Z, and global	4
Delta Displacement	X, Y, Z, and length	4
Displacement	X, Y, Z, length	4
Distance from origin		1
Position	X, Y, Z	3
Elapsed time for track		1
Elapsed time since previous picture		1
Velocity	X, Y, Z	3
Speed		1
Velocity angle with axis	X, Y, Z	3
Distance to nearest neighboring cell	1	1
Average distance to nearest neighbors	3, 5, or 9 cells	3
Mean for all *Imaris* features listed above		29
Variance for *Imaris* features listed above		29
Total displacement over window		1
Cumulative track length: Sum of displacements for all time points inside window		1
Coast coefficient: Fraction of points inside the window that are coasting (i.e. where the speed is below a fixed threshold: 1/30)		1
**Total**		**90**

### 2.7 Feature selection

The minimum Redundancy Maximum Relevance (mRMR) algorithm was used for feature selection. The mRMR algorithm follows a minimal-optimal feature selection strategy which aims to find the lowest number of relevant features for a given classification task. Compared to all-relevant feature selection strategies, the mRMR algorithm allows identifying a subset of features, which together (a combination of features) exhibit the maximum predictive performance. In this work, the F-statistic was used for relevance estimation, while Pearson correlation was used to assess redundancy. While the mRMR algorithm allows finding the best subset of features, the number of selected features should be pre-determined. In this work, the optimal number of features was chosen as part of the 3-fold cross validation, as the number for which the average cross-validation (CV) accuracy increases by less than 0.5% over 5 successive increments in the number of features. The number of selected features started from 3 and was incremented by 2 features at a time. Since the mRMR algorithm selects the most significant features first, the CV accuracy curve growth is expected to progressively slow; therefore, it makes sense to consider the growth rate as the stop criteria.

### 2.8 Classification

A balanced random forest classifier was trained to classify CD8^+^ T cell behaviors based on selected features [13]. A random forest classifier is an ensemble learning method based on the combination of multiple decision trees. The ability to fit several decision trees on various dataset subsamples allows to improve random forest’s classification accuracy while controlling for overfitting. In this work, a balanced random forest classifier was used which is a modified version of a traditional random forest classifier in which each tree is provided with a balanced bootstrap sample. Two classification strategies were evaluated: 1) Binary classification of synapse vs. kinapse behaviors; and 2) Three-class classification of dancing, poking, and scanning visually identified behaviors. For both cross-validation and test, we ensured that there was no data leakage. More specifically, data splitting was performed on an experiment basis ensuring that experiments used for training were not used for performance evaluation.

## 3. Results

### 3.1 Data distribution

Our goal was to develop a model capable of automatically classifying T cell behaviors interacting with organ specific cells. Our datasets were generated from 22 independent experiments of astrocyte: CD8^+^ T cell cocultures and 7 independent experiments of neuron: CD8^+^ T cell co-cultures. For each experiment, astrocytes, neurons, and CD8^+^ T lymphocytes were isolated from different human donors. These experiments were performed by two different experimenters (Experimenters A and B) over a three-year period. The combined experiments represented 3,284 CD8^+^ T lymphocytes tracked for 2 hours. From these tracked T lymphocytes, we extracted features directly from the *Imaris* software and compiled engineered features (mean, variance, cumulative track length, etc.) as listed in Table 1. Each entry corresponded to a given cell at a specific time step, with an interval of 1 min between successive measures. We used a 5-min sliding window (centered at the specific time step) to compute engineered features. The first dataset called Astro A contained 73,746 while Astro B had 67,127 classification samples, which were visually labeled as poking, dancing, or scanning. Poking tracks were considered as synapse-like interactions whereas dancing and scanning tracks were considered as kinapse-like interactions as we have previously published [10–12].

**Fig 2:**
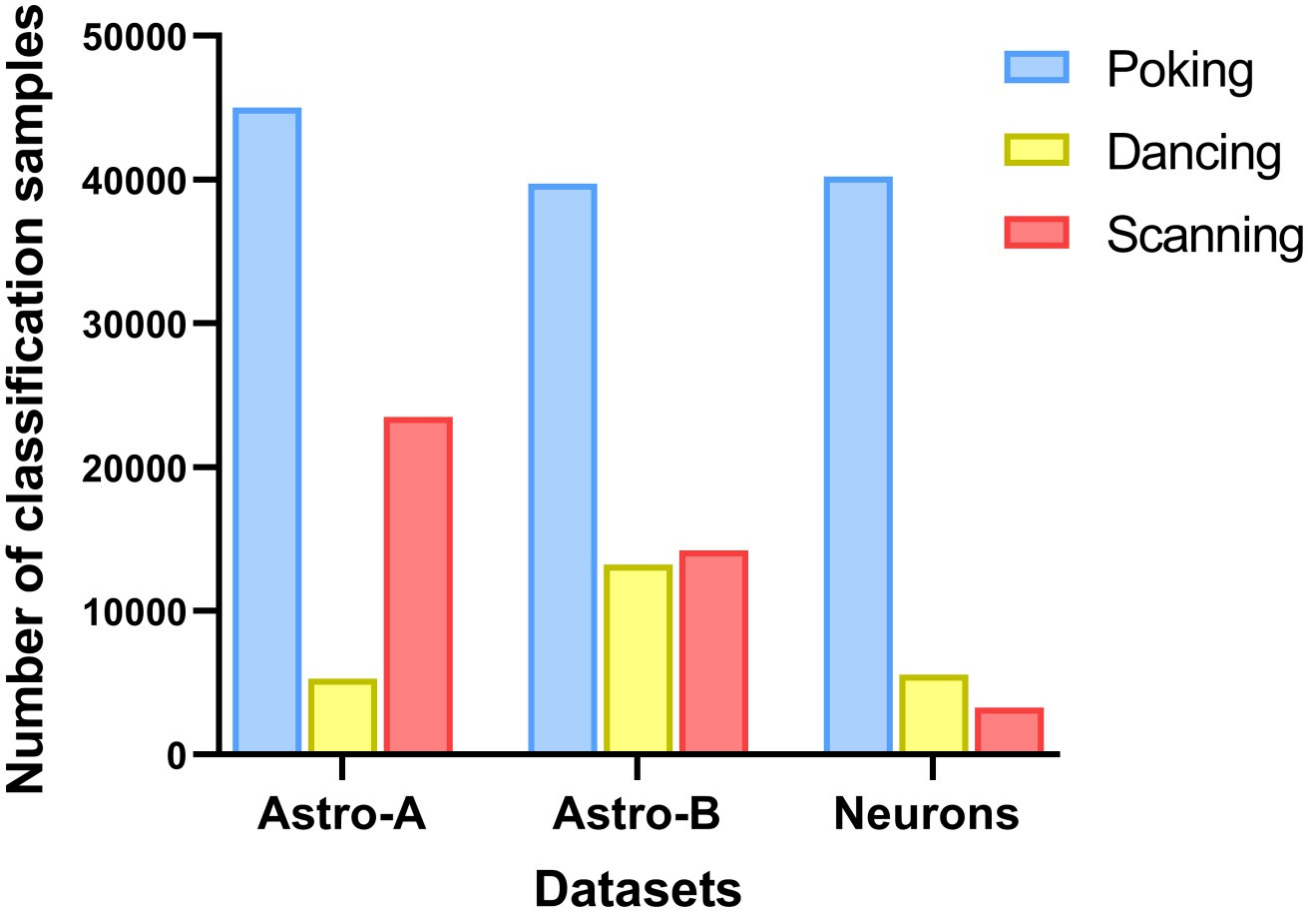
Data distribution among datasets. The number of classification samples for each dataset is shown according to the visually identified behaviors: poking (blue), dancing (yellow), and scanning (red). The total number of classification samples were 73,746, 67,127, and 49,004 for the Astro-A, Astro-B and Neurons datasets respectively.

**Fig 3:**
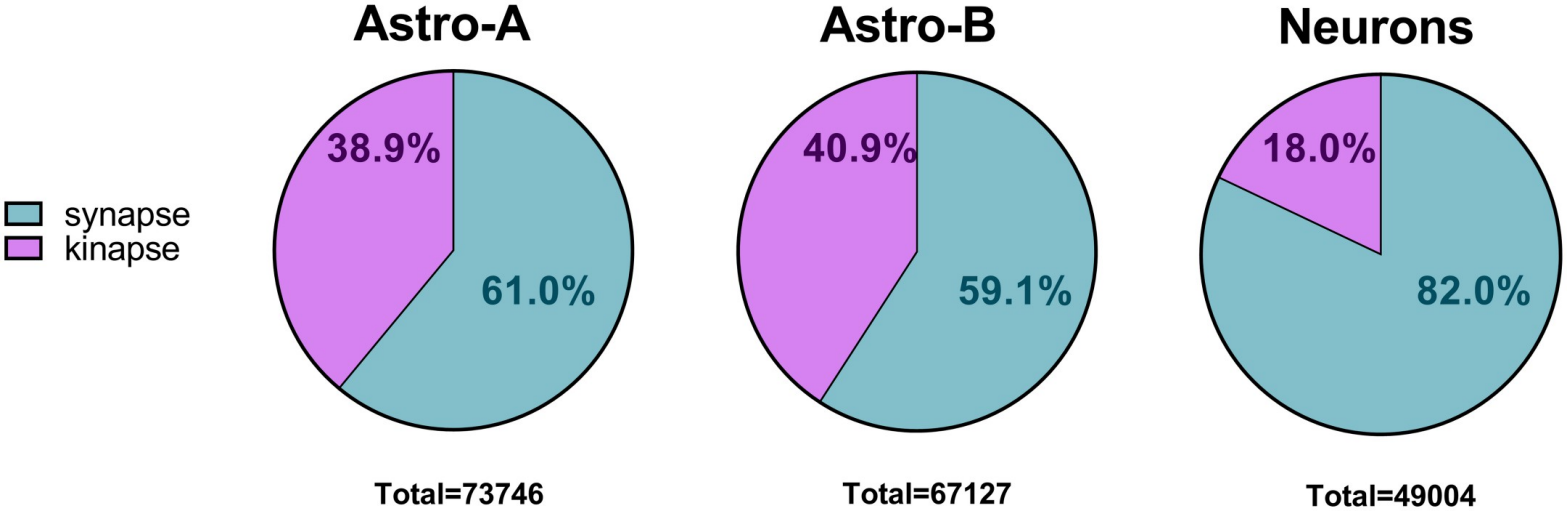
Distribution of synapse- and kinapse-like behaviors in datasets. The number of classification samples for each dataset is shown according to synapse (aqua) and kinapse-like (lavender) behaviors. The total number of classification samples is indicated under each pie chart.

In all datasets, the proportion of samples exhibiting the poking behavior was higher than for the dancing and scanning behaviors (Fig 2). Moreover, the distribution of samples into synapse-like or kinapse-like behaviors shows that these behavior classes were similarly distributed in both astrocyte datasets. In contrast, the kinapse-like behavior was less prevalent in the neuron: CD8^+^ T cell cocultures (Fig 3).

### 3.2 Feature selection

**Fig 4:**
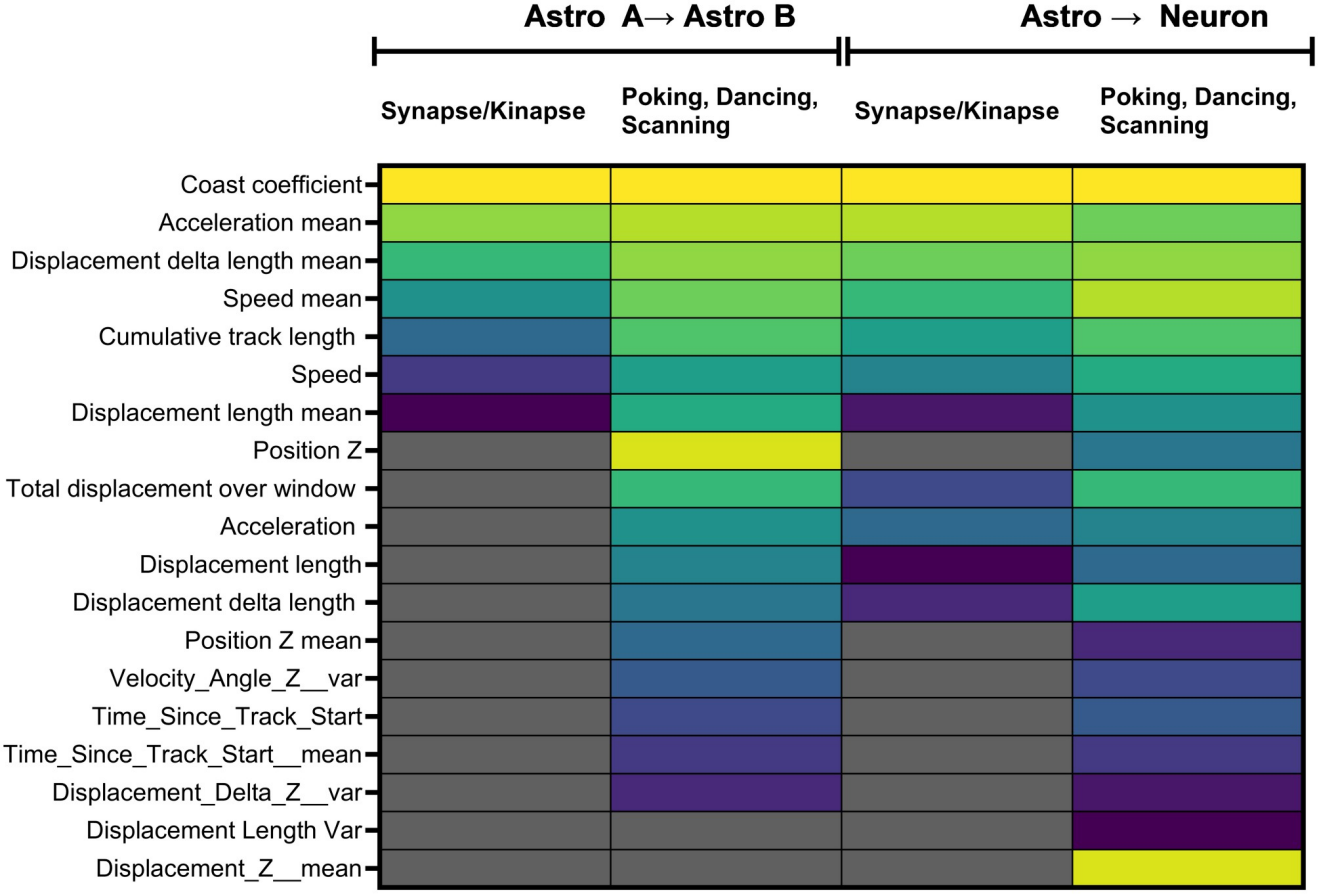
Selected features for balanced random forest for each model in decreasing importance. Features that were selected in the binary model (synapse/kinapse) or 3-class model (poking, dancing, and scanning) for the classifier trained on the Astro A dataset and tested on the Astro B dataset and for the classifier trained on the combined Astro dataset and tested on the Neuron dataset are listed from top (yellow boxes) to bottom (darker blue boxes) by order of importance. The grey boxes indicate the features that were not selected in the model.

Feature selection was performed using the mRMR feature selection algorithm (Fig 1). The optimal number of features and best features were chosen during cross validation. As expected, the classification of the three visually identified behaviors (poking, dancing, and scanning) required more features than the binary classification of synapse and kinapse behaviors. Fig 4 displays selected features in decreasing order of importance (from yellow to blue) and the number of features for different classification models. Notably, selected features were highly stable across models with the following features selected for all 4 models: coast coefficient, average acceleration, average displacement delta length, speed at current time point, average speed, cumulative track length, and average displacement length.

### 3.2 Classification

**Fig 5:**
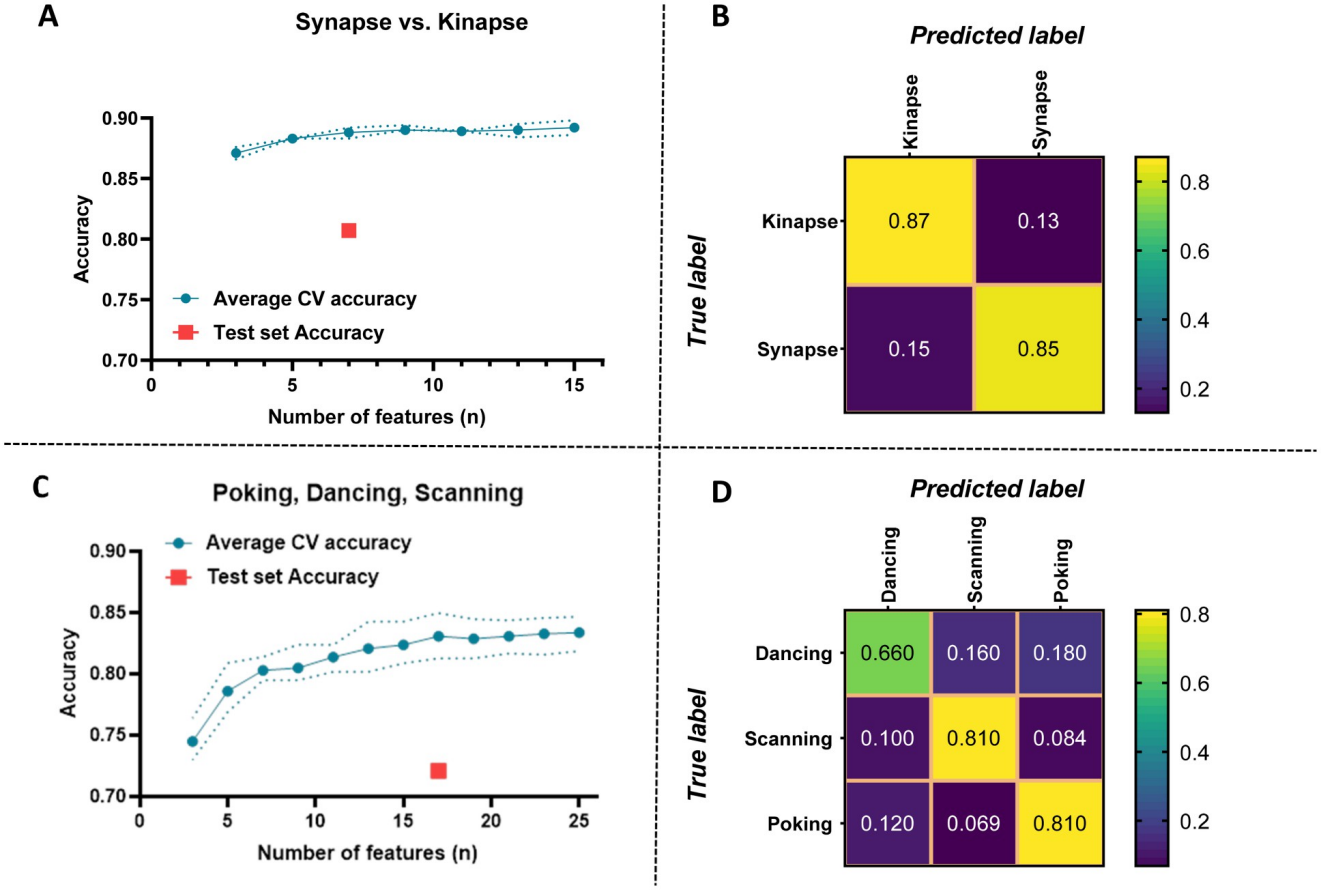
Performance evaluation of the classifier trained on the Astro A dataset and tested on the Astro B dataset. Performance evaluation of the model for classifying synapse vs. kinapse-like behaviors **(A, B)** and dancing, scanning, and poking visually identified behaviors **(C, D)** using the Astro A dataset for training and validation and the Astro B dataset for testing. **A, C)** Average cross-validation (CV) accuracy according to the number of features included in the analysis. The doted lines represent the minimal and maximal values. The test set accuracy on the Astro B dataset is indicated as a red square for the number of features selected for the final classifier. **B, D)** Confusion matrices illustrating the predicted values in the Astro B dataset using the classifier trained on the Astro A dataset for each behavior either synapse vs. kinapse **(B)** or dancing, scanning, and poking **(D)**.

To evaluate the effect of the experimenter on the performance of the classifier, the model was first optimized on data from the Astro A dataset (number of features, feature selection, training) using a 3-fold cross-validation and tested on the Astro B dataset. Fig 5 shows the performances for models optimized on the Astro A dataset and tested on the Astro B dataset. Although the held-out test set accuracy was lower than the average cross-validation accuracy (as expected), promising performances were achieved considering that the held-out test set included only data from an independent experimenter. The model achieved a held-out test accuracy of 0.81 (F1 score: 0.80 and Geometric mean: 0.81) for a binary classification (synapse vs. kinapse) using 7 features. As expected, performances were lower for the classification of the 3 visually identified behaviors achieving an accuracy of 0.72 (F1 score: 0.68 and Geometric mean: 0.70) using a total of 17 features. Selected features are shown in Fig 4.

**Fig 6:**
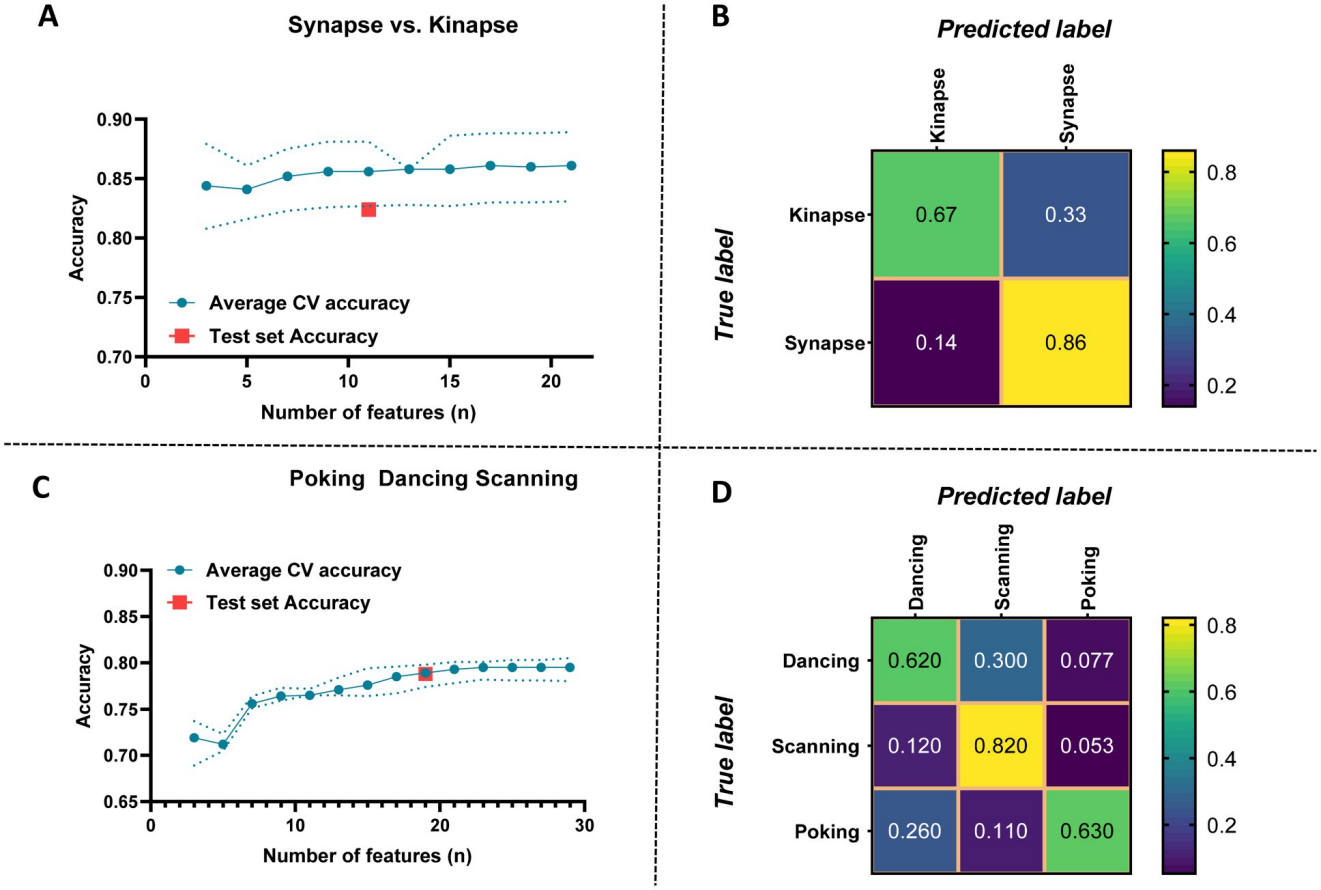
Performance evaluation of the classifier trained on the Astro dataset and tested on the Neuron dataset. Performance evaluation of the model for classifying synapse vs. kinapse-like behaviors **(A, B)** and dancing, scanning, and poking visually identified behaviors **(C, D)** using the Astro dataset (Astro A + Astro B) for training and the Neuron dataset for testing. **A, C)** Average cross-validation (CV) accuracy according to the number of features included in the analysis. The doted lines represent the minimal and maximal values. The test set accuracy on the Neuron dataset is indicated as a red square for the number of features selected for the final classifier. **B, D)** Confusion matrices illustrating the predicted values in the Neuron dataset using the classifier trained on the Astro dataset for each behavior either synapse vs. kinapse **(B)** or dancing, scanning, and poking **(D)**.

The model was then optimized on data from the whole Astro dataset and tested on the Neuron dataset. The model achieved a held-out test accuracy of 0.82 (F1 score: 0.73 and Geometric mean: 0.76) for a binary classification (synapse vs. kinapse) using 11 features. Similarly, performances were lower for the classification of the 3 visually identified behaviors achieving an accuracy of 0.79 (F1 score: 0.62 and Geometric mean: 0.68) using a total of 19 features. Selected features are shown in Fig 4. Fig 6 shows the performances for the models optimized on the Astro dataset and tested on the Neuron dataset.

### 3.3 Machine learning-selected features show segregation of behaviors using an unsupervised analysis

**Fig 7:**
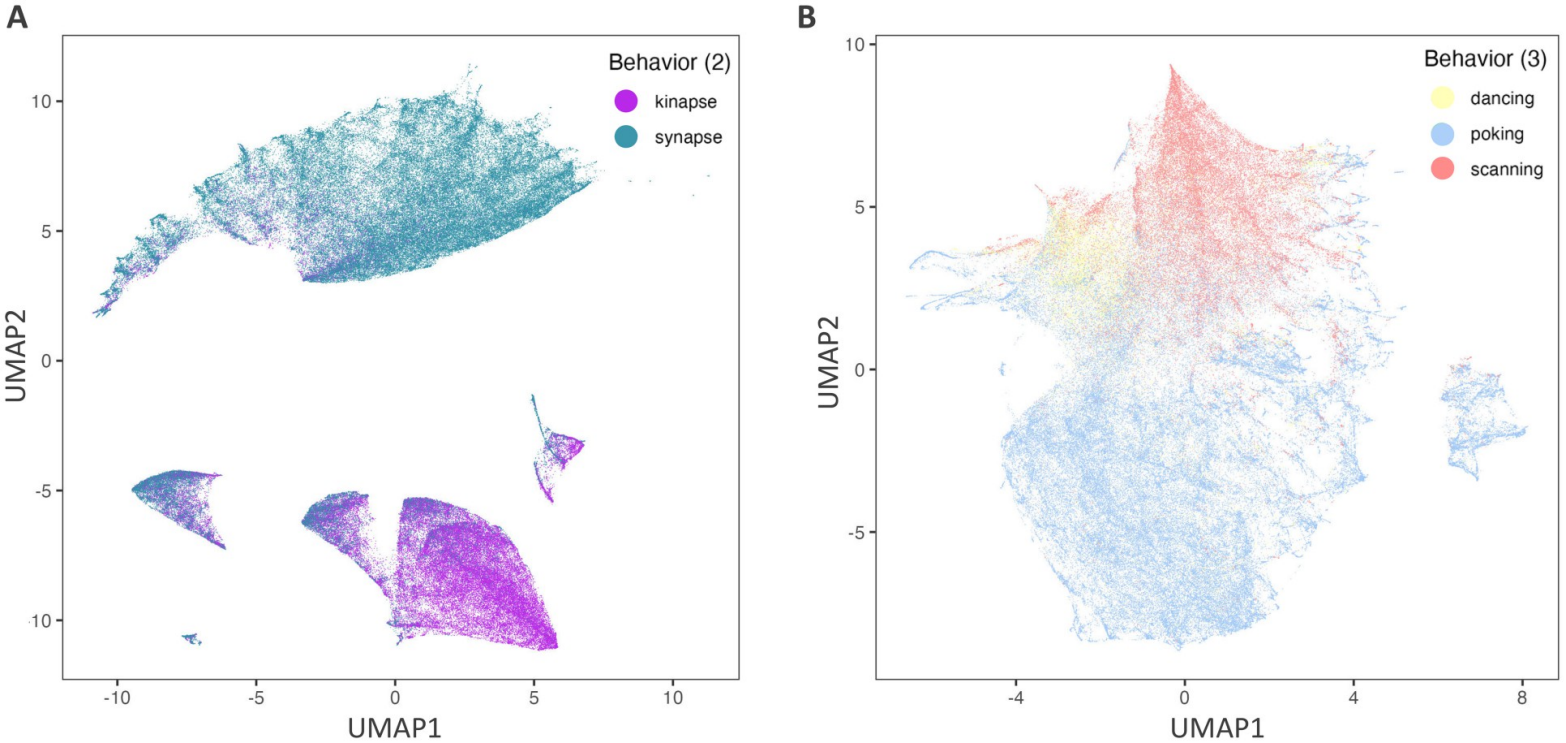
UMAP visualization of CD8+ T lymphocyte behaviors using machine learning-selected features. Features from the combined CD8^+^ T lymphocytes in Astro A, Astro B, and Neuron datasets (total of 189,877 samples) selected by machine learning either for the binary (kinapse vs. synapse-like behaviors) classifier **(A)** or for the 3-class behavior (dancing, poking, and scanning) classifier **(B)** and applied in the models in Fig 6 were used to plot on the UMAP. Selected features are listed in Fig 4.

We assessed whether the features selected by our machine-learning approach with mRMR could separate the different CD8^+^ T lymphocyte behaviors using an unsupervised approach. To provide a meaningful representation of the dataset, the Uniform Manifold Approximation and Projection (UMAP) was used as a dimension reduction technique. All three datasets (Astro A, Astro B, and Neuron) were pooled together and the 11 features of CD8^+^ T lymphocytes that were selected in the final binary model tested on the Neuron dataset (kinapse- vs. synapse-like) were used to calculate the two-dimensional coordinates of the UMAP. These features included: Coast coefficient, Acceleration mean, Displacement delta length mean, Speed mean, Cumulative track length, Speed, and Displacement length mean (Fig 4, **third column**). The vast majority of synapse-like behavior events segregated easily on the upper part of the y axis (Fig 7A). Most kinapse-like samples segregated in the bottom section of the UMAP above 0 on the x axis. The lower part of the UMAP shows smaller groups of mixed synapse and kinapse like behaviors. This UMAP confirms that the discrimination for synapse was well achieved confirming the high (0.86) classification score obtained on the confusion matrix (Fig 6B).

A second unsupervised UMAP was generated for the 3-class behaviors (poking, scanning, and dancing) on the pooled datasets using the 19 features selected in the 3-class model tested on the Neuron dataset **(**Fig 4, **last column)**. As shown in Fig 2, the poking behavior (blue) was the most abundant and segregated mainly in the bottom of the UMAP (Fig 7B). In contrast, dancing (yellow) and scanning (red) behaviors distinctly segregated in the upper part of the UMAP. At the border of each behavior cluster, samples from more than one behavior are observed, especially for dancing (yellow) behaviors, suggesting gradual transitory states between distinct behaviors. This is in line with the lower predicted value for dancing behavior (0.62, confusion matrix Fig 6D). Moreover, the UMAP reveals that the visually identified poking behavior samples (blue) contained a distinct cluster on the far-right side of the UMAP.

## 4. Discussion

Our ultimate goal is to develop relevant tools to unravel the interactions between human immune cells and brain cells under normal and pathophysiological conditions. Using time-lapse microscopy, we have previously established an in vitro co-culture model to quantify and characterize the spatiotemporal behavior of human CD8^+^ T lymphocytes while in contact with two abundant brain cell types: astrocytes and neurons [10–12]. Primary human cell cultures (CD8^+^ T lymphocytes, astrocytes, and neurons) allow modeling cell interactions as close as possible to human in vivo situations. In the current work, building on the features extracted from the broadly used *Imaris* software and subsequently engineered features (Table 1), we developed a novel classifier for the classification of CD8^+^ T lymphocyte motility behaviors (Fig 1) into either a binary classification: synapse-like vs. kinapse-like, or 3-class classification: poking, scanning, and dancing (Figs 1–4). We trained and tested the model on an astrocyte dataset from 2 experimenters (Fig 5) and tested on an independent neuron dataset (Fig 6). Finally, an unsupervised analysis of the selected features for the binary or 3-class classifier demonstrated that samples could be efficiently segregated as shown by UMAP (Fig 7), indicating that the information space yielded by the selected features permitted a clear discrimination of the cell behaviors. Our models’ stable performances show promise for expanding automated classification of cell motion to multiple experimental set-ups to avoid time-consuming manual characterization.

While previous investigations of machine learning algorithms for the analysis of cell motility behaviors have explored the possibility of detecting the presence or not of drugs [14], our proposed model was designed to classify cell motility behaviors independently of treatment, donor type, disease, and experiment. We have previously shown that in vitro treatments (e.g. cytokine treatment of astrocytes, blocking MHC class I on astrocytes, addition of a soluble NKG2D ligand) of these co-cultures modified the proportion of CD8^+^ T lymphocytes exhibiting the visually identified behaviors (poking, scanning, dancing, or synapse- vs. kinapse-like) [10–12]. Thus, improving our capacity to quantify such behaviors in an increased number of experimental set-ups was essential.

We have previously described four CD8^+^ T cell behaviors based on visual observations: scanning, dancing, poking, and round [10–12]. Scanning, dancing, and poking behaviors exhibited statistical differences between each other for most features, such as coast coefficient, track duration, speed (min, max, mean, variation), and could thus be distinctly identified. In contrast, the round behavior samples showed similar properties to the poking samples for most features [10]. Moreover, both behaviors demonstrated synapse-like characteristics; they established long-lasting interactions with neural cells [10]. While poking cells pushed pseudopods through the membrane of the interacting neural cell, round cells maintained their shape without extending pseudopods [10]; such differences could not be captured by the *Imaris* extracted features or engineered features. In future studies, the relevance of additional features, such as cell volume, could be tested to identify immune cell behaviors. For the current analysis, we combined the round and poking behaviors into one category. We developed a model using machine learning to successfully identify these three CD8^+^ T lymphocyte behaviors: poking, scanning, and dancing in different neural co-cultures (Figs 5 and 6).

The 3-class model distinguished poking (also called the synapse-like behavior) and two kinapse-like behaviors: scanning and dancing. We identified scanning CD8^+^ T lymphocytes by their flattened shape and their rapid exploring and crawling behavior [10]. In contrast, dancing CD8^+^ T lymphocytes exhibited an elongated shape oscillating around one anchor point onto the neural cell and detached themselves to anchor onto other sites [10]. Compared to the binary model (synapse vs. kinapse), the 3-class model included additional features such as Position Z (value and mean), Velocity Angle Z variation, Time Since Track Start, Displacement variation (Fig 4). The inclusion of such features took into account specific characteristics of cell movement and thus allowed the discrimination of two dynamic kinapse-like behaviors. The machine learning model confirmed our visual identification of two distinct kinapse-like behaviors: scanning and dancing. Additional investigations will be necessary to determine whether these behaviors are associated with specific T lymphocyte properties or functions. A growing number of groups are investigating the highly dynamic behaviors of T lymphocytes in the context of immune responses and pathological conditions such as HIV infection and cancer [15–19]. Although several teams have focused on the binary synapse vs. kinapse assessment, our study underlines that T cell behaviors exhibit more than two types of behaviors. Consequently, capturing and examining a large array of movement-related features will provide meaningful information. Therefore, improving our capacity to develop relevant models to assess such behaviors is deemed essential to unravel key immune responses deployed in organs.

Our machine-learning design strategy is advantageous as it allows leveraging the use of the model in other scenarios, such as different co-cultures of motile immune cells and organ specific cells. The model’s capability to maintain a satisfactory performance on more than one organ-specific cell type (astrocytes and neurons) was demonstrated by the promising performances achieved when tested on the neuron dataset. Throughout all experiments, we performed a conservative assessment of performances while ensuring no data leakage between the training, validation, and test sets. More specifically, we ensured that cells (rather than only time points) used for training were neither in validation nor in test sets.

Our work has some merit and limitations. Although the binary classification model was trained on established cell behaviors (synapse vs. kinapse), the multi-class model was trained on 3 visually identified behaviors described in our previous work [10]. While we adopted a supervised classification approach in this follow-up work, unsupervised analyses of multiple features of the interactions between CD8^+^ T lymphocytes and other cell types will provide novel insights and potentially allow the identification of additional cell behaviors that have not been described so far. Indeed, the random forest classifier’s ensemble nature makes it less interpretable than simple clustering and machine learning models. However, the mRMR feature selection algorithm allowed us to investigate which features were the best in terms of relevance and redundancy. In addition, the random forest classifier allows assessing selected features’ importance. S1 and S2 Figs show features’ importance in terms of mean decrease in impurity, as considered in the final model, for the binary and 3-class classifications, respectively.

Interestingly, the performance of our models was maintained using datasets acquired from multiple human cell donors (for CD8^+^ T lymphocytes, astrocytes, and neurons) and different experimenters (experimenter A and experimenter B). Our machine learning models demonstrate that artificial intelligence-based tools can accelerate and improve the identification of normal or dysfunctional interactions of human T lymphocytes with different organ-derived cells in physiological and pathological contexts. In addition, it should be noted that the proposed method relies on the manual correction of cell tracks identified using the automated detection of the IMARIS software in cases of misdetection or non-detection. While this approach is broadly used by the scientific research community, it can be laborious and prone to human error or bias. Given that this algorithm is embedded in the IMARIS software, it was not possible to investigate possible improvement strategies. However, future versions of the IMARIS software with upgraded automated tracking methods could reduce the need for manual corrections of cell tracks.

While our model shows promising performances, we propose that future improvements could mitigate the remaining 20% of uncertainty. For instance, the current version of the classifier provides the certainty (prediction probability) of each label, which can be further explored to detect possible misclassifications. Our tests show that the average probability for the predicted label is lower on misclassified cells than on correctly classified cells. Thus, flagging predictions below a certain probability threshold for manual review by one user could substantially improve the classification rate on the remaining cells. Further work can be done to quantify this potential improvement and develop a threshold selection algorithm to balance the need for human review with the prediction quality. Moreover, the detection of transient classification changes in a predicted sequence (isolated prediction for a very short duration) and the development of a heuristic correcting the prediction based on preceding and subsequent labels could decrease the uncertainty. In the current work, we implemented a moving window approach with a 5-minute track duration. While proposed engineered features incorporate temporal information, a fixed-duration window might not capture all relevant information, particularly for interactions of varying duration. Alternatively, multi-scale sliding windows could be investigated to enhance the performance. Finally, it should be noted that while the balanced random forest algorithm automatically under-samples the training data by providing each tree with a balanced bootstrap sample, its performance could still be impacted by class imbalances, particularly the lower number of “dancing” samples. However, we tried an over-sampling strategy but it did not improve the results. Future work could investigate if “dancing” cells exhibit a transitory behavior that makes them intrinsically harder to classify.

### Summary

T lymphocytes migrate into organs and interact with local cells to perform their functions. The mechanisms whereby human T lymphocytes communicate with organ-specific cells and contribute to pathobiological processes remains largely unresolved. Infiltration of T lymphocytes into the central nervous system (brain and spinal cord) is associated with multiple neurological disorders. Therefore, to characterize the behavior of human T lymphocytes reaching the human brain, we performed time-lapse microscopy on human CD8^+^ T lymphocytes co-cultured with either primary human astrocytes or neurons. Using traditional manual and visual assessment of microscopy data, we identified distinct CD8^+^ T lymphocyte motility behaviors. However, such characterization takes time and effort. In this work, we trained and validated a machine-learning model for the automated classification of behaviors of CD8^+^ T lymphocytes interacting with astrocytes and neurons using datasets generated from two different experimenters and 29 independent experiments performed over a 3-year period. A balanced random forest was trained for the binary classification of established classes of cell behaviors (synapse vs. kinapse) as well as visually identified behaviors (scanning, dancing, and poking). Feature selection was performed during 3-fold cross-validation using the minimum redundancy maximum relevance algorithm. Results show promising performances when tested on a held-out dataset of CD8^+^ T lymphocytes interacting with astrocytes with a new experimenter and a held-out independent dataset of CD8^+^ T lymphocytes interacting with neurons. When tested on the independent CD8^+^ T cell-neuron dataset, the final model achieved a binary classification accuracy of 0.82 and a 3-class accuracy of 0.79. This novel automated classification approach could significantly reduce the time required to label cell motility behaviors while facilitating the identification of interactions of T lymphocytes with multiple cell types. Finally, unsupervised analysis of the features selected by machine learning showed that they allowed the segregation of samples into the three different behaviors (scanning, dancing, and poking) or two classes of behaviors (synapse-like vs. kinapse-like).

## Supporting information

S1 Fig Features’ importance in the final binary classification model based on mean decrease in impurity. (TIF)

S2 Fig Features’ importance in the final 3-class classification model based on mean decrease in impurity. (TIF)
