# Supplementary figures and images for: Classification of T lymphocyte motility behaviors using a machine learning approach
Source: PLoS Comput Biol. 2023 Sep 11;19(9):e1011449. doi: 10.1371/journal.pcbi.1011449 (PMC10513376; doi:10.1371/journal.pcbi.1011449)

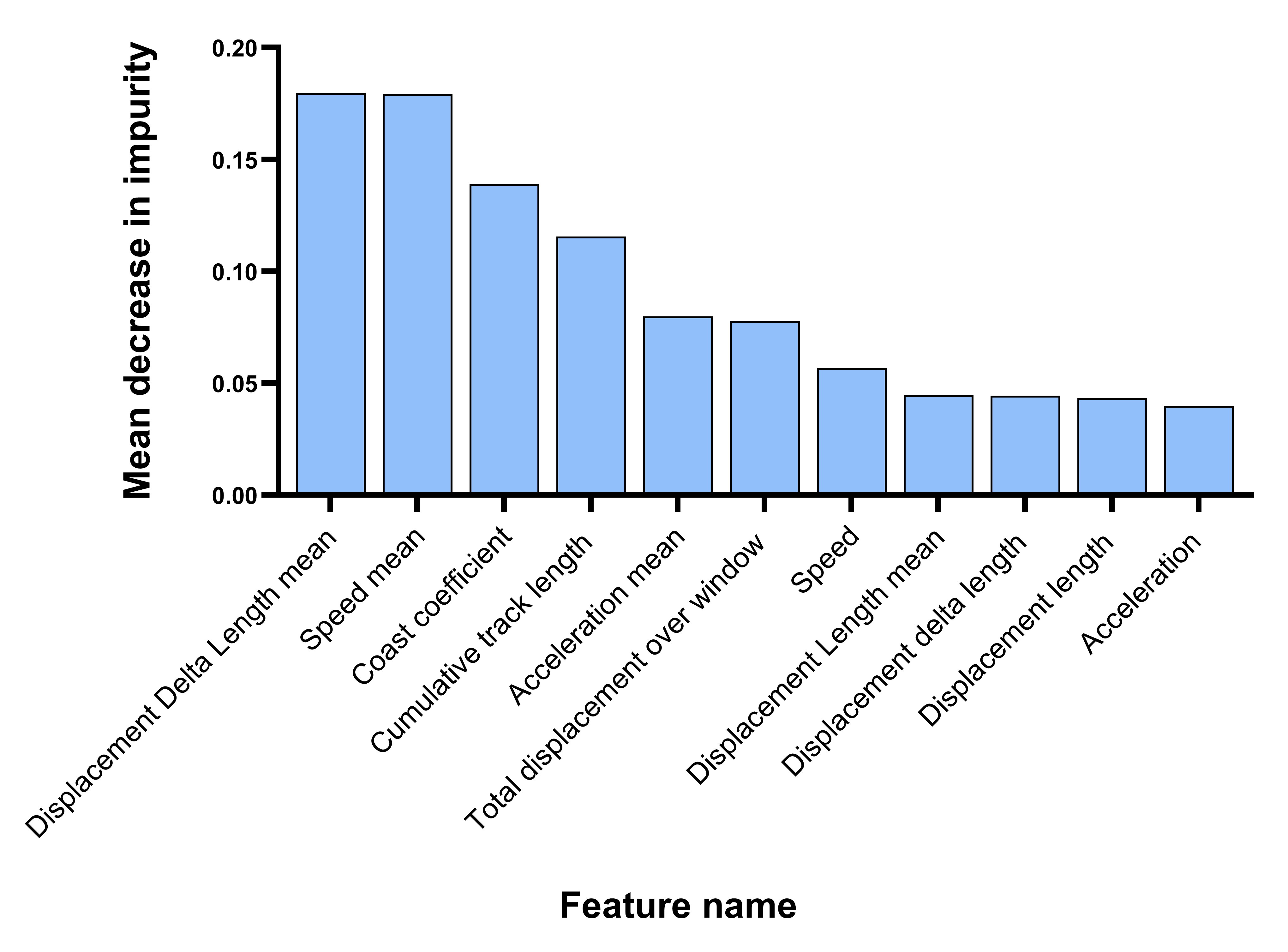

Supplement: S1 Fig — (TIF) [file pcbi.1011449.s001.tif]

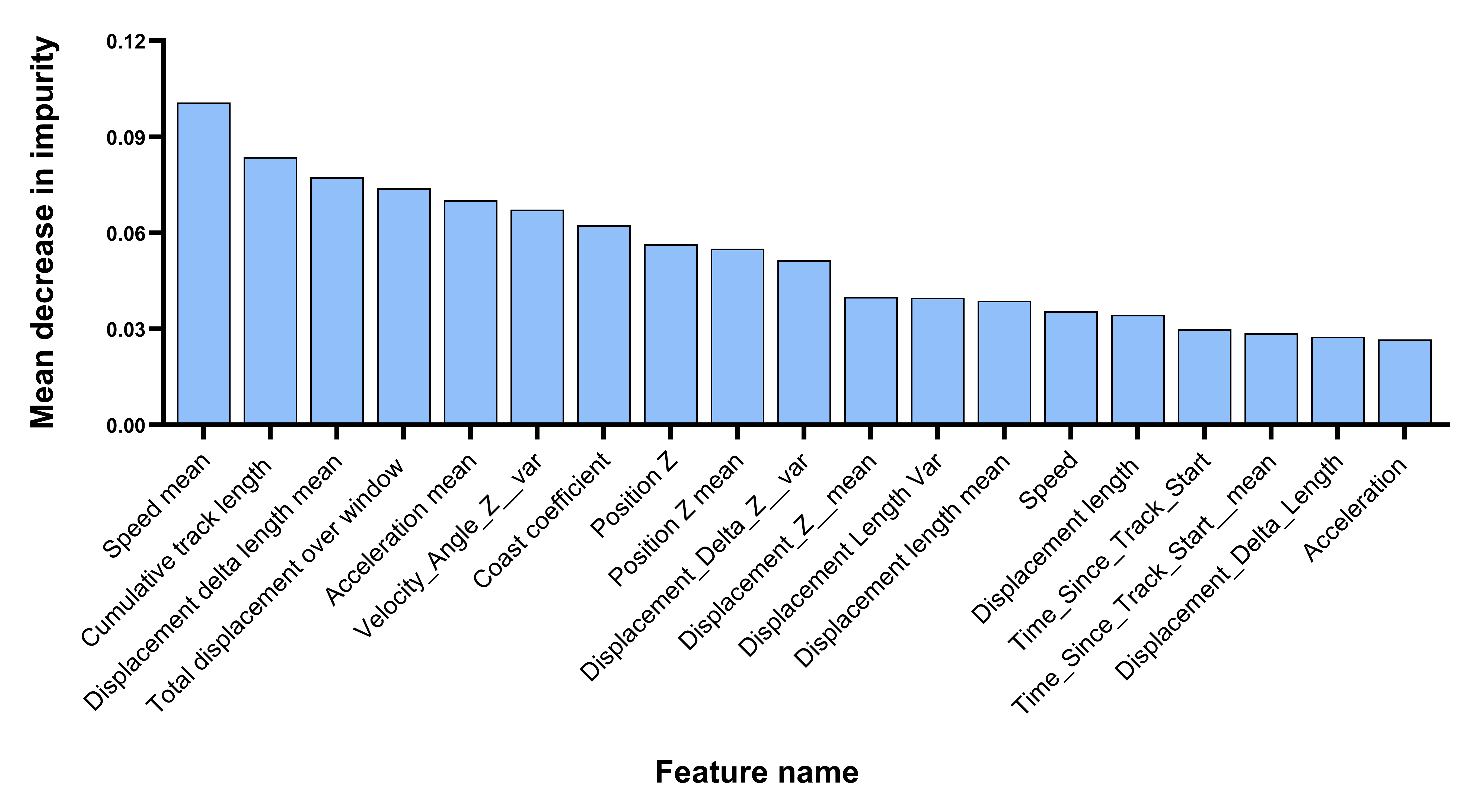

Supplement: S2 Fig — (TIF) [file pcbi.1011449.s002.tif]
